# Ulcerative Colitis with Superior Sagittal Sinus Thrombosis: A Case Report

**DOI:** 10.31729/jnma.8216

**Published:** 2023-07-30

**Authors:** Somika Basnet, Abashesh Bhandari, Bhupendra Kumar Basnet, Sabin Thapaliya

**Affiliations:** 1 Helping Hands Community Hospital, Chabahil, Kathmandu, Nepal; 2 Department of Internal Medicine, Nepal Medical College and Teaching Hospital, Jorpati, Kathmandu, Nepal

**Keywords:** *case reports*, *inflammatory bowel diseases*, *ulcerative colitis*

## Abstract

Ulcerative colitis is a chronic inflammatory, idiopathic, condition of the mucosa of the colon. Cerebral venous thrombosis is one of the serious and rare complications of inflammatory bowel disease. We report a case of a 27-year-old female patient with complaints of loose stool 5 to 6 episodes per day mixed with blood for 10 days. The patient has severe per rectal bleed, not controlled despite adequate measures and a few days later developed altered sensorium and fits. This case highlights prompt diagnosis and early treatment managing both severe ulcerative colitis and sagittal venous thrombosis. Immediate recognition of her disease improved her condition drastically and increased her survival.

## INTRODUCTION

Ulcerative colitis (UC) is a chronic inflammatory, idiopathic, condition of the mucosa of the colon.^1^ It starts in the rectum and extends proximally in a continuous manner through the entire colon and presents as bloody diarrhoea. It is characterised by intestinal as well as extraintestinal manifestations.^2^ It is an emerging problem in the healthcare field of Nepal and affects the young age group of 30-40 years.^3^ Incidence of overall systemic thromboembolic events in UC patients ranges from 1-7.7% but sagittal thrombosis is not so common.^4^ Patient usually presents with neurological symptoms like headache and seizure.^5^

## CASE REPORT

A 27-year-old female patient presented to a tertiary care hospital in Kathmandu with complaints of loose stool 5 to 6 episodes per day mixed with blood for 10 days. She also gave a history of multiple episodes of vomiting mixed with food particles, non-projectile and non-bilious. She also had a history of fever for 12 days not associated with chills and rigour was relieved on taking medication and maximum temperature was not recorded.

Her history, family history and psychosocial history were insignificant. Examination findings showed pallor. On lab workup, her haemoglobin level was 9 g/dl, a total leucocyte count (TLC) of 4580/mm^3^ with increased neutrophil 95%. Stool routine and microscopy showed plenty of pus cells, red blood cells 5-6/hpf and a cyst of *Entamoeba histolytica* was isolated.

A suspicion of UC was made and a colonoscopy was performed which showed ulcerative colitis. A computed tomography (CT) scan of the abdomen was performed which showed findings consistent with diffuse colitis. CT also showed the presence of a linearly oriented, non-enhancing area suggestive of thrombosis partially occluding the lumen of the right, middle, and left hepatic vein at a distance of approximately 2.5 cm from the inferior vena cava. Partial thrombosis was also evident at the branch supplying segment VI of the right portal vein.

**Figure 1:**
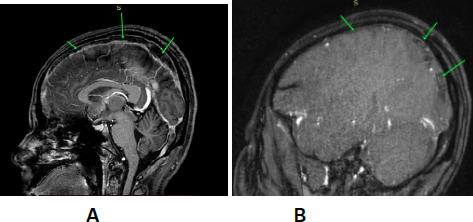
MRI of the brain with MRV A) showing loss of normal blood flow in the entire superior sagittal sinus, B) acute thrombosis with venous infarction of bilateral frontal lobes.

The patient developed seizure activity while she was in treatment and magnetic resonance imaging (MRI) of the brain with magnetic resonance venography (MRV) was done which showed acute thrombosis in the entire superior sagittal sinus, right transverse/sigmoid sinus and medial half of the left transverse sinus. Venous infarctions in the bilateral frontal lobes (right >left) and right parietal lobe with subcortical punctate haemorrhage was seen. Hematoma with subarachnoid extension in the area of right frontal venous infarction was seen. MRI of the brain with magnetic resonance venography (MRV) (Figure 1).

The patient was classified as a case of severe UC with superior sagittal sinus thrombosis and the patient was started on heparin, enema and steroid. The patient was started on azathioprine after 3 days of treatment and was started on adalimumab on the eleventh day of diagnosis after which her symptoms decreased. Heparin was changed to an oral anticoagulant later. She responded well to medicine with no apparent side effects. On her subsequent follow-up, she improved her clinical symptoms with control of her bleeding per rectum with no further deterioration of the sensorium and other neurological complications.

## DISCUSSION

Chronic inflammation and idiopathic ulcerative colitis affect the colon's mucosa. It manifests as bloody diarrhoea, which is the disease's defining symptom. Due to the disease's alternating remission and exacerbation episodes, its clinical history is unpredictable and characterised by intestinal and extraintestinal symptoms.^1,2^

In a prospective study done in Nepal from 2017 to 2020 AD, UC was identified in 352 patients from 7423 colonoscopies thus the incidence of UC in the context of Nepal was calculated as 23.7 per 1000 colonoscopies per year.^3^

Thrombotic complications such as superior sagittal sinus thrombosis in patients with inflammatory bowel disease require improved awareness and prevention.^4^ Superior sagittal sinus thrombosis is one of the types of dural venous sinus thrombosis. The patient often presents with symptoms of headache which is the most common symptom, seizures, hemiplegia, quadriplegia or paraplegia, visual disturbances, and nuchal rigidity.^5^ The most common site of thrombotic complications in inflammatory bowel disease (IBD) patients are deep vein thrombosis (DVT) and pulmonary embolism (PE), other sites such as the mesenteric vein, portal vein and retinal veins are also affected, however, the incidence of sagittal sinus thrombosis is low among the involved sites.^6^

Cerebral venous thrombosis in ulcerative colitis was first noted in a study done in 1966.^7^ From then till now only a few cases of cerebral thrombosis in ulcerative colitis have been noted to date. Diagnosis of UC should trigger the assessment of thrombosis risk. For this thorough history taking of the patient should be done and an evaluation of the risk of thrombosis should be performed, assessment of estrogen intake or use of estrogen-containing contraceptives should be taken in female patients, and a family history of the patient for any thrombotic risk should also be assessed.^2^ In our case patient was diagnosed as having an active flare of ulcerative colitis and was managed accordingly then after a few days she developed a seizure and was diagnosed as having sagittal sinus thrombosis.

The study also mentions that very few reported clinical cases have presented with cerebral sinus thrombosis in a patient suffering from ulcerative colitis. Sagittal sinus thrombosis can be best diagnosed using cerebral angiography or MRV.^5^ Absent flow void is seen within the affected venous sinuses which can also be visualised as the delta sign. According to a study MRI and MRV with or without contrast is the gold standard measure for diagnosing sinus thrombosis.^8^

It is studied that spontaneous platelet aggregation and hyperhomocysteinemia are seen more frequently in patients suffering from IBD and thus can be the reason for thromboembolic complications in a patient suffering from UC. Also, the coagulation cascade is altered in IBD patients which includes increased prothrombin fragments, increased fibrinogen, and increased factor V, VII and VIII.^9^ Increase in coagulation factors helps in clot formation and as a result, there is increased thrombosis risk. Patients suffering from IBD also have impaired systemic fibrinolytic capacity which could also be a contributor to the thrombotic phenomenon.^10^ A study has concluded that trials conducted with subcutaneous heparin or low molecular weight heparin were shown to be effective in the treatment of thromboembolic phenomena in active ulcerative colitis.^8^

This is an uncommon case of sagittal sinus thrombosis seen in a case of ulcerative colitis. Prompt diagnosis and early intervention could drastically change the disease outcome and improve overall survival. Our patient was treated with subcutaneous heparin right after the event of diagnosis and was on longterm anticoagulation for further prevention of such complications again. We also conclude that physicians should be prompt in diagnosing thromboembolic phenomena like sinus thrombosis in cases of ulcerative colitis who develop neurological symptoms during the active stage of ulcerative colitis and inactive stage as well.

## References

[1] Ordas I, Eckmann L, Talamini M, Baumgart DC, Sandborn WJ (2012). Ulcerative colitis.. Lancet..

[2] Al Ghadeer HA, Alsalman SA, Alobaid J, Abdi ZI, Aljereish SS, Buhlaiqah S (2022). Cerebral venous sinus thrombosis is a reversible complication of ulcerative colitis.. Cureus.

[3] Paudel MS, Khanal A, Shrestha B, Purbey B, Paudel BN, Shrestha G (2021). Epidemiology of inflammatory bowel diseases in Nepal.. Cureus.

[4] Zitomersky NL, Verhave M, Trenor CC (2011). Thrombosis and inflammatory bowel disease: a call for improved awareness and prevention.. Inflamm Bowel Dis..

[5] Absher JR, Madeline L, Webb SW, Rayes M (c2018). Reference module in neuroscience and biobehavioral psychology..

[6] Deskur A, Zawada I, Blogowski W, Starzyska T (2019). Cerebral venous sinus thrombosis in a young patient with ulcerative colitis: a case report.. Medicine (Baltimore)..

[7] Yerby MS, Bailey GM (1980). Superior sagittal sinus thrombosis 10 years after surgery for ulcerative colitis.. Stroke..

[8] Martin-Masot R, Perez PO, Nieto JS, Leon MM, Martinez AP, Blasco-Alonso J (2019). Central venous sinus thrombosis in a boy with acute severe ulcerative colitis.. Front Pediatr.

[9] Chiarantini E, Valanzano R, Liotta AA, Cellai AP, Fedi S, Ilari I (1996). Hemostatic abnormalities in inflammatory bowel disease.. Thromb Res..

[10] Gris JC, Schved JF, Raffanel C, Dubois A, Aguilar-Martinez P, Arnaud A (1990). Impaired fibrinolytic capacity in patients with inflammatory bowel disease.. Thromb Haemost..

